# Improving motorcyclist safety through hearing in all directions: survey results concerning a novel protective helmet with earpieces

**Published:** 2024-07

**Authors:** Felipe Morales V., Yixin Wang, Ken C. Pohlmann, Rodrigo Posada, Adolfo Vargas, Jose M. Ramirez, Alejandra Bejarano, Eric Anderson, David C. Schwebel

**Affiliations:** ^*a*^ Resonar, Pereira, Colombia.; ^*b*^ Department of Psychology, University of Alabama at Birmingham, USA.; ^*c*^ School of Music, University of Miami, USA.; ^*d*^ Seguros SURA, Bogota, Colombia.; ^*e*^ Vroom Network, Carson City, Nevada, USA.

**Keywords:** Motorcycle helmets, Safety, Injury, Crash, Hearing, Acoustics

## Abstract

**Background:**

Every year over 200,000 motorcyclists are killed globally. One poorly understood risk factor for motorcycle crashes is the role of motorcyclists’ ability to hear traffic and other sounds around them in all directions. Most motorcycle helmets protect the head and permit vision in the forward direction, but they impair the wearer’s ability to hear. This study evaluated user perceptions of Protective Helmet with Earpieces Equipped, a novel system that affixes technology onto existing motorcycle helmets to allow motorcyclists to better hear the surrounding environment in all directions.

**Methods:**

59 Colombian traffic police agents who rode motorcycles daily completed self-report surveys about their traditional motorcycle helmet, used a Protective Helmet with Earpieces Equipped helmet for two months, and then completed a follow-up survey. We tested two hypotheses: (a) at follow-up compared to baseline, motorcyclists would report decreases in perceived safety, comfort, and ability to detect sounds with their traditional helmet, as well as increased fatigue and stress from motorcycling with their traditional helmet, and (b) at follow-up, motorcyclists would report high perceived safety and perceived importance of hearing traffic sounds in all directions while motorcycling. Wilcoxon signed-rank test evaluated hypotheses.

**Results:**

Participants rated perceived safety while using their traditional helmet to be significantly lower after using the Protective Helmet with Earpieces Equipped compared to before using it (Z=-3.5, p less than .001). There were no changes in other variables assessed. Following use of the new helmet, participants reported greatly improved safety (M=4.56, SD=0.54, where 4=safer and 5=substantially safer) and perceived the importance of hearing traffic sounds in all directions as high.

**Conclusions:**

Improved auditory perception could increase motorcyclist safety. Participating traffic agents felt the Protective Helmet with the Earpieces Equipped greatly improved their ability to hear and im-proved their motorcycling safety. Perceived safety while using their traditional helmet decreased following use of the alternative.

## Introduction

Road traffic injuries constitute a global public health problem, killing approximately 1.19 million people annually.^1^ An estimated 20-25% of those deaths, or about 200,000 fatalities, are to motorcyclists.^1-2^

Most global road traffic injury burden occurs in low- and middle-income nations, especially among vulnerable road users like motorcyclists. In Colombia, the middle-income nation where the current research was conducted, about 60% of deaths related to road traffic collisions are to motorcycle riders.^3^ About 4,800 Colombian motorcyclists were killed in 2022, equivalent to more than 13 deaths per day in a country of 51.5 million people.^3^ Many more motorcyclists suffer serious injuries, and the human and economic toll on families is enormous.^4-5^

A wide range of driver, motorcyclist, and environmental factors contribute to motorcycle crash risk.^6-7^ One factor that is comparatively understudied but potentially critical is the sensory capacity of the motorcycle operator. The safest and most widely used motorcycle helmets enable adequate vision in all necessary directions, but generally restrict hearing. Unlike human visual perception, which is most acute only in the direction the viewer is facing, aural perception functions in a 360° environment. Auditory perception allows a listener to grasp what is happening not just in front of them, but also behind and to their sides.

When operating a motorcycle, the ability to hear in all directions, including to the side and behind, may offer safety benefits.^8^ Published research on the role of auditory perception for safe motorcycling is limited, but related work suggests pedestrians use listening to protect their safety when crossing streets.^9-11^ In the three most-common causes of motorcycle crashes at intersections – the motorcyclist making a left-turn at a T-intersection, the motorcyclist being struck by a vehicle going straight on a cross-road while passing a 4-way intersection, and the motorcyclist being struck by a vehicle turning left on a cross-road while passing a 4-way intersection^12^ – the motorcyclist would likely benefit from hearing traffic sounds from vehicles on their left and right. Related research reports on efforts to understand and improve acoustic awareness of vehicle traffic and reduce wind-related air flow noise for motorcyclists,^8,13-14^ concluding that improved auditory perception could allow motorcyclists to detect risks, take emergency aversive action, and avoid collisions.

**Figure 1:**
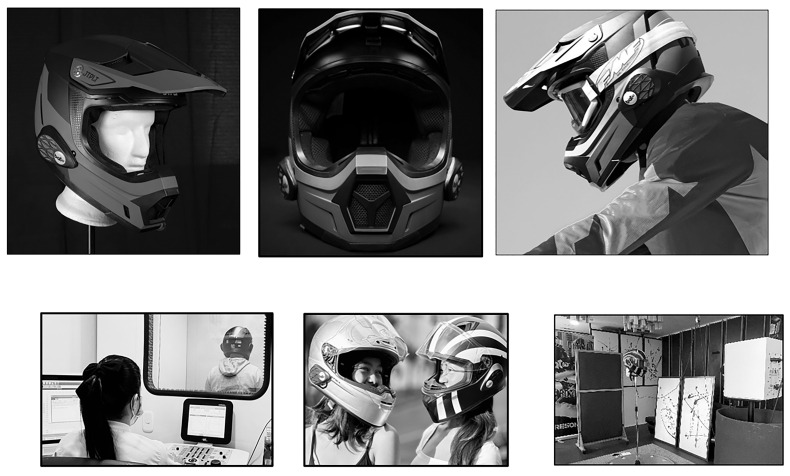
Photographs of the Protective Helmet with Earpieces Equipped System by Resonar

Despite the likely value of auditory stimuli for motorcycling safety, most currently-marketed motorcycle helmets are designed primarily to absorb and reduce impact to the head. They permit vision in the front-facing direction but they restrict hearing, especially to the side and behind. Recently, new technology, called Resonar, was developed and patented to resolve this issue (Protective Helmet with Earpieces, patent CO2017006010A1, 2017, CO US 2018 AU SI KR PL HU CN PT WO EP MX BR CA RS MY HR PE RU JP ES 2019 PH IL ZA). These novel devices are affixed to currently-marketed motorcycle helmets and facilitate 360° hearing to the wearer, similar to the way they would hear if riding helmetless, without changing or sacrificing other safety features of the helmet (See Figure 1). From an engineering perspective, the earpiece devices accomplish this through a three-dimensional audio reference system that receives sound impulses, identifies their spatial location and distance, and then transduces the analog signals through concentric cones toward the driver’s ears.

The present study was designed to collect initial information on motorcyclists’ perception of the novel Protective Helmets with Earpieces Equipped, including their assessment of improved safety while using an equipped helmet compared to their traditional helmet and their perceived importance of hearing in all directions while motorcycling after using equipped helmets. We posited two primary hypotheses: (a) the motorcyclists would report decreased perceived safety, comfort, and ability to detect sounds with their traditional helmet, as well as increased fatigue and stress from motorcycling with their traditional helmet, after using the equipped helmet, and (b) the motorcyclists would report high levels of perceived safety and perceived importance of hearing traffic sounds in all directions while motorcycling after using the equipped helmet. We also explored whether any reported safety increases were associated with participants’ age, sex, daily motorcycle helmet use, self-rated motorcycling expertise, or self-rated road rule expertise.

## Methods 

Participants. Through convenience sampling, we recruited 59 traffic police agents from four Colombian cities, Dosquebradas, La Virginia, Manizales, and Pereira, to participate. The sample included 39 men (66%) and 20 women (34%) and was an average of 39.27 years old (SD = 10.12). All conducted their traffic police work using motorcycles. Nine participants (15%) completed the first research survey but not the second. All participants provided consent to respond to the surveys, and analysis of anonymized data was approved by the Institutional Review Board at University of Alabama at Birmingham.

Protocol. After consenting to study participation, traffic police agents completed a short self-report survey (detailed below) and then were introduced to the novel helmet technology. They were given a well-fitting, US Department of Transportation-certified helmet with Resonar earpieces equipped to use for two months. Following those two months, they completed a second brief self-report survey.

Baseline measures. Demographic information was collected at baseline, including participant age and sex. We also asked about hours of daily helmet use (answered on a 5-point scale: less than 1 hour a day, 2 to 3 hours a day, 3 to 4 hours a day, 4 to 5 hours a day, more than 5 hours a day), years of experience riding motorcycles (5-point scale: less than 1 year, 1 to 3 years, 3 to 5 years, 5 to 7 years, and more than 7 years) and self-reported knowledge about road signs and traffic regulations (5-point scale: none, very little, moderate, strong, expert). 

Measures assessed in both surveys. We considered five outcomes that were assessed both at baseline and after participants had used the protective helmet with earpieces equipped for 2 months. Three related to perceptions of their traditional helmet: perception of the helmet’s safety, perception of the helmet’s comfort, and perception of their ability to hear sounds in all directions. These ratings were made on a four-point scale (none, average, high, very high).

The remaining two questions assessed physical state after motorcycling with their traditional helmet. The first asked about fatigue and the second about stress while riding. They also were answered on a four-point scale (always, almost always, hardly ever, never). Responses were reverse-coded for analysis so that higher scores indicated higher levels of fatigue and stress. 

Measures assessed following equipped helmet use. Six items were included in the second survey to assess perceptions of the helmet after its use. Four focused specifically on perception of sound with the helmet, asking participants to rate how important it was to hear traffic sounds, motorcycle sounds, sounds on the left/right in front of them, and sounds on the left/right behind them. These 4 items were answered on a 5-point scale, from unimportant to very important.

The last two items asked of participants assessed perceived safety of the protective helmet with earpieces equipped following use of it for two months. One read, “During the time you used the Resonar-equipped helmet, how safe did you feel compared to using your traditional helmet?” Participants answered on a 5-point scale (substantially safer, safer, indifferent, no improvement, or got worse), with responses reverse-coded so that higher scores indicated greater perceived safety. The second read, “Do you think a helmet device like Resonar that allows listening to the surroundings with better perception of sound sources in all directions would help avoid collisions while riding a motorcycle?” Responses were provided on a 5-point scale, from “completely agree” [5] to “completely disagree”.^1^

Data analysis. Survey responses were transferred into Excel and then SPSS for analysis. First, we considered safety-related questions asked both at baseline and following use of the Resonar protective helmet with earpieces equipped for 2 months. Because most distributions were non-normal, we analyzed change over time using Wilcoxon Signed Rank Test. A sensitivity analysis was conducted using parametric t-tests. Next, we considered descriptive data of perceptions collected following use of the earpiece-equipped helmet. Last, we considered correlations between demographic variables and perceived safety measures.

## Results

Table 1 shows descriptive data and Wilcoxon Signed Rank Test results for the items asked both at baseline and after using the equipped helmet. As shown, the perceived safety of riding with the traditional helmet was significantly lower after using the helmet with earpieces equipped (M = 2.56, SD = 0.71) than it was prior to using the new helmet (M = 2.95, SD = 0.70; Z = -3.5, p <.001). Contrary to expectations, there was no significant change in perceived comfort of the traditional helmet (Z = -1.75, p = .08) or the perceived ability of the traditional helmet to detect sound directionality and distance (Z = 0.14, p = .89). There also were no changes in fatigue or perceived stress experienced while motorcycling (Z = 0.95, p = .34 and Z = 0.91, p = .36, respectively). Table 1 also shows results of the parametric t-tests computed as a sensitivity analysis to the Wilcoxon tests. Results were highly similar.

**Table 1:** Descriptive characteristics of outcome variables, Wilcoxon test results, and results of sensitivity t-test

Outcome Variables	Baseline M (SD)	Post M (SD)	Wilcoxon Signed Rank Test		Sensitivity t-test (df=49)	
			Z	p	t	p
**Perceived safety with traditional helmet**	2.92 (0.70)	2.56 (0.71)	-3.50	<.001	4.06	<.001
**Perceived comfort of traditional helmet**	2.78 (0.89)	2.60 (0.88)	-1.75	0.08	1.81	0.08
**Perceived ability to detect sound directionality and distance with traditional helmet**	2.25 (0.82)	2.14 (0.86)	0.14	0.89	1.34	0.19
**Fatigue experienced while motorcycling**	2.29 (0.77)	2.44 (0.81)	0.95	0.34	-0.94	0.35
**Perceived stress experienced while motorcycling**	2.14 (0.96)	2.28 (0.95)	0.91	0.36	-0.88	0.38

N=50.

Table 2 displays descriptive data concerning the traffic police agents’ perception of the earpieces-equipped helmet. As shown, they perceived significant improvement in safety when using the new helmet (M = 4.56, SD = 0.54, where 4 = safer and 5 = substantially safer) and strongly felt that a helmet device that helped them hear surroundings in all directions would help avoid collisions while motorcycling (M = 4.78, where 4 = agree and 5 = completely agree). The participants also provided high ratings concerning their perception of the importance of hearing traffic sounds (M = 4.38, SD = 0.83), motorcycle sounds (M = 4.44, SD = 0.79), sounds to the left and right in front of them (M = 4.44, SD = 0.71), and sounds to the left and right behind them while motorcycling (M = 4.52, SD = 0.61).

**Table 2:** Perceptions of safety and importance of hearing relevant sounds after using novel protective motorcycle helmet with earpieces equipped (N=50)

Perceptions	M (SD)
Perceived safety improvement with novel helmet	4.56 (0.54)
Perceived ability of helmet device like Resonar to help avoid collisions	4.78 (0.42)
Perceived importance of hearing traffic sounds after using novel helmet	4.38 (0.83)
Perceived importance of hearing motorcycle sounds after using novel helmet	4.44 (0.79)
Perceived importance of hearing traffic sounds on both sides in front of me while motorcycling, after using novel helmet	4.44 (0.71)
Perceived importance of hearing traffic sounds on both sides behind me while motorcycling, after using novel helmet	4.52 (0.61)

Table 3 summarizes bivariate correlations between perceived changes in safety, demographic, and other relevant variables. Perceived improvement in safety while using the earpieces-equipped helmet was significantly associated with sex (r = -.36, p = .01); women perceived less improvement in safety than men. It was also associated with perceived road rule expertise (r = .29, p = .04), with participants perceiving they had greater expertise about road rules also perceiving greater improvement in safety using the new helmet. Finally, we detected significant associations between higher self-reported expertise in road rules and greater change in perceived safety of the traditional helmet before and after using the earpieces-equipped helmet (r = 0.30, p = 0.03).

**Table 3:** Correlations between demographic measures and perceived safety with novel earpieces-equipped helmet

	M (SD)	2	3	4	5	6	7
**1. Age (years)**	39.27 (10.12)	-0.17	0.00	0.33*	0.31*	0.19	0.08
**2. Sex (Male=0, Female=1)**	34% female	-	-0.08	-0.30*	0.06	-0.36*	-0.18
**3. Average daily helmet use (5-point scale)**	3.53 (1.60)	-	-	0.24	0.25	0.03	0.04
**4. Motorcycling experience (5-point scale)**	4.53 (1.02)	-	-	-	0.03	0.04	-0.15
**5. Perceived road rule expertise (5-point scale)**	4.17 (0.38)	-	-	-	-	0.29*	0.30*
**6. Perceived safety improvement with novel helmet (5-point scale)**	4.56 (0.54)	-	-	-	-	-	0.27
**7. Change in perceived safety of traditional helmet, after using novel helmet (Difference score, 4-point scale)**	0.42 (0.73)	-	-	-	-	-	-

* p <0.05.

## Discussion

Motorcycling is dangerous, killing over 200,000 people annually worldwide.^1-2^ Effective prevention strategies are wide-ranging,^15^ but have traditionally overlooked the relevance of auditory perception in all directions. With the capacity to hear what is beside and behind them, sensory capacity that is largely absent with traditional helmets,^14^ motorcycle operators may be able to take aversive action to reduce collisions.

This study offers the first published data on user perception concerning novel protective helmets equipped with earpieces to facilitate hearing in all directions while motorcycling. Results from the sample of traffic agents who ride motorcycles daily for occupational purposes are compelling; they reported considerable improvement in perceived safety while using earpieces-equipped helmets instead of their traditional helmet. They felt they could hear better, and concordant with previous literature,^8,13-14^ they felt that hearing better in all directions was important to their safety.

The participants also judged the perceived safety of riding with their traditional helmet to diminish after using the novel helmet. They did not perceive any change to the comfort of their traditional helmet, or to the fatigue or stress experienced while motorcycling with their traditional helmet, suggesting that the novel helmet components did not reduce comfort or impact fatigue or stress while riding. The correlation analyses we conducted indicated that perceptions about the earpieces-equipped helmet were similar for all subgroups we studied, although there was some indication that men perceived greater improvement in safety than women and that participants who felt they had greater knowledge about road rules also perceived greater improvement in safety.

Efforts to improve motorcycling safety must be multi-faceted.^15^ As is true in other injury prevention domains, efforts to address one predictive factor causing motorcycle injuries are likely to be less effective than efforts that concurrently address multiple predictive factors.^16-17^ Continued work to engineer safer roadways, alter car and truck driver behavior, and enforce both general road regulations as well as motorcycle-specific regulations such as helmet laws, are essential. Use of helmets equipped with earpieces to facilitate auditory perception in all directions might add one more tool to our arsenal of strategies to achieve safer motorcycling worldwide. Results from this study offer excellent promise, and we recommend continued research to evaluate and disseminate use of helmets with earpieces equipped to improve motorcyclist auditory perception. In particular, future research might extend beyond self-report surveys to consider crash data; do users of equipped helmets have reduced crash risk? Randomized trials are recommended as well, demonstrating change in perception among randomly-assigned groups of motorcyclists using helmets with earpieces equipped versus those who retain use of their traditional helmet.

Like all research, this study had limitations. Many of the survey response results had restricted ranges, as perceptions tended to be strong. This is a positive finding, in that users rated the earpieces-equipped helmets highly, but it impacted our ability to detect subgroup differences due to the restricted range of variables. Also, each outcome was measured only through a single item; future research might incorporate multi-item scales to better assess outcome constructs of interest. Also limiting was the sample, which was derived conveniently and entirely from individuals operating motorcycles for occupational purposes in a single middle-income country, Colombia. Extension in the future to recreational riders, larger and more representative samples, and to individuals in lower-income countries is recommended.

In conclusion, our results offer evidence that novel motorcycle helmets equipped with earpieces that facilitate hearing in all directions might improve riders’ ability to hear stimuli around them, increasing rider safety and reducing crash risk without sacrificing comfort.
